# Health economics and health preference concepts to orthopedics practitioners

**DOI:** 10.1590/1413-78522014220200456

**Published:** 2014

**Authors:** Carlos Delano Mundim Araújo, Daniela Francescato Veiga, Bernardo Hochman, Luiz Eduardo Felipe Abla, Neil Ferreira Novo, Lydia Masako Ferreira

**Affiliations:** 1 Universidade do Vale do Sapucaí, Pouso Alegre, MG, Brazil, Universidade do Vale do Sapucaí, Pouso Alegre, MG, Brazil; 2 Universidade Federal de São Paulo, São Paulo, SP, Brazil, Universidade Federal de São Paulo, São Paulo, SP, Brazil

**Keywords:** Quality of life, Quality-adjusted life years, Cost-benefit analysis

## Abstract

The aim of this study was to describe concepts of health economics in order to update and provide the orthopedic practitioner decision making parameters based on preferences. Four basic types of studies of economical evaluation were presented (cost minimization analysis, cost-benefit, cost-effectiveness and cost-utility), as well as the origin, the concept, advantages and disadvantages of using QALY and utility. It was discussed the importance of costs and of SF-6D, an instrument able to get through the utility data from the Medical Outcomes Study 36-item Short Form Health Survey (SF-36). Physicians, especially orthopedic practitioners, are increasingly using technologies which are progressively expensive, thus, they should be able to understand health economics concepts, the importance of utility in clinical decision making process and economic analysis in health.09+

## INTRODUCTION

Health care policies for the population are a challenge faced by many countries, including Brazil. The United States are currently facing a health crisis, with cumulative costs summing up two trillion dollars per year, the equivalent to 16% of the country Gross Domestic Product. Resources are scarce and finite, either in human, time, financial, physical and structural terms; and all choices must be made every days.^1^ Therefore, the need for studies to assess the economic impact of health initiatives has recently grown.^2^

The economic evaluation is important for decision-making regarding the allocation of resources, aiming greater efficiency and effectiveness in its use.^3^ Without careful analysis of all aspects involved in an intervention, including costs and consequences, wrong decisions may eventually be adopted in practice.^2^ Research in the field of quality of life have led clinicians, scientists, economists and managers to become interested by the impact brought by new technologies in terms of health status and costs for the health systems.^4^

The development of science and technology has improved the effectiveness of diagnostic and therapeutic methods. This, in turn, increases life expectance, but increases the costs of health systems.^4^ The orthopedist is seen as a consumer of expensive, fragile and difficult to maintain technologies being massively harassed by advertisements, which invariably leads to a cost which is transferred to customers, leveraging social and individual cost of orthopedic medicine to ever higher and elitist levels.^5^

Decision making that are based on preferences for health states, along with the economic analysis focused on the patient's perspective, improve the distribution of finite resources in the face of a growing and increasingly challenging demand.

This study aims to contextualize these principles that should be understood, studied and used by orthopedists.

### Types of economic assessment studies 

There are four basic types of studies for economic evaluation: cost minimization analysis, cost- benefit, cost-effectiveness and cost-utility. The costs of health interventions are expressed in monetary units. Cost-minimization indicates which alternative has the lowest cost. Cost- benefit generate a quotient between resources spent and acquired benefits.^2^

The analysis of cost- effectiveness enables full economic evaluation in health, with effects expressed in clinical and epidemiological units.^6^ In these studies, the effectiveness can be assessed through quality of live instruments.^7^ Cost- effectiveness also allows checking which strategy is more effective.^8^

Cost-utility analysis establishes a relationship between the amount spent and the quality and quantity of life gains. A cost-utility analysis refines the cost-effectiveness outcome, it allows to compare any types of health interventions and their effects, measured in Quality-adjusted life years (QALY).^6^ This assessment can show alternatives to maximize achieving the target within a minimum budget.^8^ For an instrument be able to provide objective data for cost-utility assessment, it must be based on preferences.

The perspective of the analysis is the point of view from which the analysis is conducted, and it may be the patient's perspective, the program's perspective (a service provider), the payer's perspective (a health insurance plan) or the society's perspective. Cost-utility studies can be seen as a cost-effectiveness economic analysis conducted from the patient's perspective that takes as a parameter the QALYs' clinical effectiveness.^4^

### Preference as a parameter for decision making

It is important that the physician is able to measure the perception of the patient to assess the benefit of his interventions. Current medicine is not only concerned with the objective data of a medical intervention, but also with the patient's opinion about the procedure he underwent. The assessment of quality of life and its domains is difficult to perform, since these concepts include subjective elements.

By the need to assess these subjective questions arose indexes, instruments, or indicators (questionnaires with scores) able to measure and compare these aspects anywhere in the world. Since the validation of various questionnaires until demonstrating their efficiency, the scientific community increasingly seeks to assess satisfaction and patient response to different situations and interventions in medicine.

A large number of instruments, methods and techniques have been proposed to assess the quality of life and its components of patients with various diseases. These instruments can be divided into generic and specific. Generic are those applicable to a wide variety of people because they include aspects of function, dysfunction, and emotional distress. Generics have two subcategories, developed to measure all important aspects of quality of life related to health (health profile) and those that reflect patient preferences for a specific health condition or "utility".

There is a tendency that studies based on patient preferences are used in economic decisions and resource distribution.^4^ There are two types of preferences: utility or usefulness, which is obtained under conditions of uncertainty, and value, which is obtained under conditions of certainty.^1^

### Utility

Utility is a specific type of preference, measured under conditions of uncertainty, according to the paradigm established by von Neumann-Morgenstern.^4^ It represents the soundness of the preference of an individual under conditions of uncertainty that is represented in numerical form from zero to one, where zero corresponds to death and one perfect health in the space of one year.

Utilities are obtained by individual values ​​and may represent a group, by the sum of measurable and comparable individual utilities.^9^ The advantages of obtaining utility: being based on preferences, it enables the calculation of QALY, allows comparison of different interventions and structure the tree decision.

Utility can be obtained directly or indirectly. There are basically three well-established techniques for the direct measurement of utility (i.e., that argues the individual directly on his preference).^10^ ^,^ ^11^ It consist of performing the valuation (weights system) of a large and representative sample of the normal population. They are: the choice by chance (Standard Gamble), the choice by time (trade-off time) and the visual analogue scale (VAS).

The choice by chance (Standard Gamble) is the most accurate method of measuring preference.^1^ The only technique capable of capturing the utility-type preference is the choice by chance, also known as true utility. The other techniques capture the value type preferences. The Standard Gamble have been recommended in studies reviewing the different approaches to the measurement of preferences.^12^

It asks respondents to choose between two alternatives. In one of the alternative it is offered an intermediary state of health (e.g., a chronic low back pain), under certainty conditions. Alternatively, two health statuses are offered in condition of uncertainty in case the patient undergoes a therapeutic procedure: a state of health would be the best possible (with a P probability likely to occur) and it would be the worst case, for example death (with probability 1-P).

This measurement is necessary to determine the indifference point p* (point at which the patient is indifferent between treatment A or B), because this indifference point p* is the utility that the patient attributed to his current health status in a scale ranging from 0 to 1.

The choice by time (time trade-off) measures the value type preference (in certain conditions) and utility that is considered false. Two options for conducting in certainty conditions are given, for the patient to decide facing a chronic disease. The patient must answer how many years of life he is willing to give in exchange for avoiding a particular chronic condition.

The time trade-off method is a practical alternative to the Standard Gamble.^13^ The values ​​obtained by these techniques correspond to preferences for different health status in the general population, specific for each region.

The visual analogue scale (VAS) also measures the value type preferences (in certainty conditions). A line is drawn on one end equivalent to, while the other extremity equals one. The value one can correspond to the best possible health status, and zero the worst possible (e.g., death).

Direct preference measurements are generally complex, time-consuming, costly, and are conducted with the assistance of visual resources. In order to obtain the valuation of a normal population, a very large sample, time, human and financial resources are needed.^14^ The biggest cost for obtaining valuations of health status is related to data collection.

The cognitive ability, aversion to risk and numerical skills can affect the measurement by Standard Gamble, an effect known as construct irrelevant variance. Still, these tools have been considered as the most suitable for the cost-utility analyzes, due to be more theoretical than based on facts.^12^

The utilities can be measured indirectly through questionnaires with multi-attribute systems for various health status based on preferences. Among the best known instruments are: Quality of Well-Being Scale, Health Utilities Index (HUI), EuroQol-5D, and the Short-Form 6 Health Survey Instrument (SF-6D).^15^

One way to test the properties of indirect measurements consists in administering an indirect method in conjunction with a direct method of preferences valuation, such as Time Trade Off or Standard Gamble, and to examine the degree of convergence between them.

In Brazil, the congruence between the indirect methods (based on questionnaires) and the direct methods was tested, and the method that had the highest number of significant correlations was the SF -6D,^15^ being also the only one that correlated significantly with the Standard Gamble technique.^16^ SF -6D is derived from the world's most widely used generic instrument for assessing quality of life, Medical Outcomes study 36-item Short Form Health Survey. SF - 36 has a multitude of data available in the literature and has been translated, adapted and validated for use in Brazil.^16^

Another way to test the stated preferences is to analyze the degree of convergence between the values generated by different indirect measures of choice, such as SF -6D, EQ- 5D or HUI.^11^ For the cost-effectiveness/utility analysis, indirect measures have been preferred because they allow in a fast and objective manner the use of social values​​, obtained throughout the community.

### Quality-adjusted life years (QALY)

The concept of QALY was developed in the 1970s from studies on chronic renal failure.^17^ However, the term "quality-adjusted life years" (QALY) was first used only in 1976. Currently it is interpreted as the result of merging of dimensions quality and quantity of life, expressed in the form of utility.^9^

An extensive review published in 1992 included 51 economic evaluations using QALY as a measure of outcome.^18^ Still, QALY outcome raised doubts until after 1996, when convincing studies about its effectiveness emerged, and it has become widely accepted as a reference standard in economic studies.^3^

QALY has not been designed for decision making for a single patient, although sometimes it has been placed on clinical decision analysis. It is based on the premise that individuals change their health status over time and each health status has a value.

QALY, as a reference unit , expressed in the form of utility, can be used for cost-utility analyzes in health, because it provides a standard measure to compare disease and programs in assessment to incorporate health technology.^4^ The great advantage of this approach is that it provides a standard measure to compare diseases and programs in evaluations to incorporate health technology.

Currently, QALYs are used in most economic evaluations by many regulatory agencies, which have made the cost-effectiveness assessment as part of their process of decision making. United States (U.S. Panel on Cost Effectiveness in Health and Medicine) and Great Britain (Institute of Health and Clinical Excellence, NICE) have endorsed the conventional use of QALY as standardized method to promote comparative cost -effectiveness of different health interventions.

Some limitations of QALY would be prejudice towards older people, to give low priority to those patients who are responsible for their diseases, giving highest priority to patients who have dependents, be benevolent towards patients from lower social class (with exceptions for male subjects), and have no preference as to the distribution of benefits, unless they are small, where the preference falls into the situation that it is better to give it to a few people.

Regardless of their methodological controversies the study of cost-effectiveness, cost-utility, QALY and utility are important allies for economic evaluation of health technology and their principles should be understood by health administrators.

## DISCUSSION

The managerial knowledge of costs by health professionals, especially physicians, is strategic to the development of successful cost system and for its economic analysis factor. However, the use of economic evaluations in surgical procedures is still scarce and perhaps this is due to the training deficiency of the surgeon.^19^

Studies of cost-effectiveness and in particular cost-utility have been growing worldwide, often sponsored or supervised by government agencies, in order to guide the manager regarding the allocation of resources.^3^ Mistakenly, the terms cost -effectiveness and cost utility have been used interchangeably, although having different concepts.^7^

Authors argue that the cost-effectiveness of an intervention simply mean that, when comparing this intervention to another one similar, this or that would be more efficient or would require less investment for the same final outcome.^14^ Assessment of cost-effectiveness have been established as the predominant valuation techniques in health economics from 1979 being more used to assess diagnostic tests and cost-benefit evaluations ​​in preventive interventions.^14^

Much of the studies cost-benefit and cost-effectiveness is achieved with medication, due to sponsorship of pharmaceutical laboratories. Conducting a systematic review/meta-analysis should be a key step in the study of cost-effectiveness. These are additional reasons for the scarcity and difficulty in performing these studies by surgeons.

The cost-effectiveness and cost utility analysis were included among the top five references to resource allocation by directors of private health plans, although they are not as used by resources managers.^14^ Therefore, the economic evaluation in health has a prominent role and requires managers facing new challenges in the ongoing quest for efficiency and effectiveness of activities.

Despite criticism, cost analyzes try to bring more efficiency in resource allocation. The limitation of resources used in the provision of medical care and the increasing demand of health needs justify studies and practices in this area. This diversity of opinions about the cost analysis is understandable and perfectly justifiable, since it does not seem possible that in such a vast field it can exist as an instrument to satisfy all authors, enabling the achievement of desired results in any kind of study.^10^

One of the largest studies of limiting costs is a difficult generalization to other countries. The difference between currency values, prevalence and odds between countries, especially when comparing developed countries to developing countries, limits the applicability of these studies.^20^ Therefore, it is important to perform analyzes within Brazil, with data reflecting the country's reality.

Although most health expenditures in Brazil come from Supplementary medicine, approximately 75 % of the population depends exclusively on the public health care system.^20^ Resources from SUS (Brazilian Unified Health System) represent a significant portion of public hospitals financing and its by-procedure pay chart does not allow full coverage of the costs of a hospital. Is has been estimated in 1997 that each hospital exceeds on a daily basis, on average, 60% of the amount provided by SUS.^21^

In fact, the amount paid by SUS reflects the reality of Brazil, a large country with large distortions. On the one hand, there is low pay for basic medical procedures and medical fees. On the other hand, suppliers of orthopedic implants, stents and image tests are well paid. This distortion could contribute to indications of questionable treatments and surgeries.

For these reasons, in Brazil, cost studies and economic analyzes that give special attention to procedures performed by the public health care system should be encouraged in order to assist SUS managers in the proper allocation and distribution of resources.

Utility, as part of a cost and economic analysis study should be obtained more frequently to evaluate medical interventions. A special relevant importance is presented by SF- 6D, an instrument able to get through the use of data from SF-36. SF -6D has had its measure properties tested and validated for use in Brazil in its original format, and it is the only derivative of Standard Gamble, considered the most consistent in health analyzes.^16^

## FINAL CONSIDERATIONS

However, for this to happen it is necessary that physicians better understand some economics concepts, as well as the importance of utility as a tool capable of accomplishing the structuring of clinical decision analysis and economic analysis in health. The orthopedist has a prominent position in these cases, due to the gradual and progressive use of expensive technologies, and as a commercial target of large enterprises.
